# Beta-amyloid Deposition in Biliary Atresia Reduces Liver Regeneration by Inhibiting Energy Metabolism and Mammalian Target of Rapamycin Signaling

**DOI:** 10.14309/ctg.0000000000000536

**Published:** 2022-09-22

**Authors:** Xinbei Tian, Ying Wang, Ying Zhou, Bo Wu, Ying Lu, Jun Du, Weipeng Wang, Wei Cai, Yongtao Xiao

**Affiliations:** 1 Division of Pediatric Gastroenterology and Nutrition, Xin Hua Hospital, School of Medicine, Shanghai Jiao Tong University, Shanghai, China;; 2 Shanghai Institute for Pediatric Research, Shanghai, China;; 3 Shanghai Key Laboratory of Pediatric Gastroenterology and Nutrition, Shanghai, China;; 4 Department of Pediatric Surgery, Xin Hua Hospital, School of Medicine, Shanghai Jiao Tong University, Shanghai, China.

## Abstract

**INTRODUCTION:**

Biliary atresia (BA) is a devastating obstructive bile duct disease found in newborns. This study aims to investigate the roles and involved mechanisms of beta-amyloid (Aβ) in the pathogenesis of BA.

**METHODS:**

We examined the distribution of Aβ protein and its precursor in the livers of patients with BA. A murine liver organoid and a zebrafish model were established to investigate the exact roles of Aβ in liver regeneration for BA.

**RESULTS:**

Both Aβ mRNA and protein significantly increased in livers of infants with BA and deposited around the central vein. In the plasma, Aβ elevated significantly in patients with BA and positively correlated with liver injury progression. *In vitro*, Aβ treatment induced abnormal morphology and caused impaired growth in liver organoids. Energy metabolism analysis demonstrated Aβ increased aerobic glycolysis and reduced ATP synthase in organoids, in which the mammalian target of rapamycin signaling was suppressed. *In vivo*, Aβ42 exposure caused liver degeneration in zebrafish larvae.

**DISCUSSION:**

Aβ depositing in livers of infants with BA reduced the liver regeneration through attenuating mitochondrial respiration and mammalian target of rapamycin signaling.

## INTRODUCTION

Biliary atresia (BA) is a serious liver disease caused by the obstruction of bile ducts, which leads to progressive liver fibrosis (1). Prompt diagnosis facilitates early Kasai portoenterostomy to restore bile drainage (2). Still, nearly half of the patients show no improvement in bile drainage after Kasai portoenterostomy (3,4); thus, liver transplantation turns to be the final solution for most patients (5). However, insufficient understanding of the underlying pathogenic mechanisms of BA has led to slow progress in the field of therapy.

β- and γ-secretases produce beta-amyloid peptide (Aβ) through proteolytic processing of the transmembrane protein amyloid beta (Aβ) precursor protein (APP). The accumulation of Aβ in the brain is believed to be an early toxic event in the pathogenesis of Alzheimer disease (6). Aβ has 2 most common isoforms, Aβ 1–40 and Aβ 1–42. Aβ 1–40 accounts for about 90% of the total secreted Aβ and is the main soluble Aβ in biological fluids (7). Aβ 1–42 easily forms aggregates *in vivo* and is believed to play an important role in neurodegeneration (8,9). Aβ 1–42 has higher cytotoxicity and is more directly related to pathology of neurodegenerative diseases (10). Studies have found that Aβ inhibits respiratory chain function, and in cells lacking functional respiratory chain, Aβ toxicity seems to be reduced (11,12). Several studies have also shown that exposure to Aβ leads to abnormal mitochondrial function in neurons and other cellular populations within the brain (13–15). The accumulation of Aβ around the bile ducts in BA livers was observed in recent researches (16); yet, its pathobiological roles are still unknown.

By combining organoids and zebrafish, the pathobiology of Aβ on livers of patients with BA was explored in this study. We found that Aβ increased and deposited in the livers of patients with BA. Aβ inhibited the growth of liver organoids and development in zebrafish larvae. Furthermore, Aβ treatment would suppress the mitochondrial respiration and mammalian target of rapamycin (mTOR) signaling.

## METHODS

### Human specimens

A total of 34 plasma samples were obtained from patients with BA before surgery. A total of 22 plasma samples from choledochal cysts (CC, n = 15), diaphragmatic hernia patients (n = 3), atrial septal defect (n = 3), and cholangitis (n = 1) were considered as the non-BA group. All plasma samples were obtained before operations. Among them, 5 liver specimens from patients with BA undergoing surgery and 5 liver tissues taken from CC were used for immunofluorescence and immunohistochemistry. All tissues were obtained during operations. All patients' guardians provided written informed consent. Patients' characteristics are presented in (see Supplementary Table S1–S4, http://links.lww.com/CTG/A881). This study was approved by the Faculty of Medicine's Ethics Committee of Xin Hua Hospital (XHEC-D-2022-028).

### Animal studies

The 6- to 8-week-old C57BL/6J mice used in this study were obtained from Jihui Laboratory Animal Care (Shanghai, China). The zebrafish were raised in Shanghai Institute for Pediatric Research zebrafish facility. All animal experiments were approved by the Shanghai Jiao Tong University School of Medicine–affiliated Xin Hua Hospital Animal Care and Use Committee (XHEC-F-2022-053).

### Statistical analyses

All experiments were repeated at least 2 times with identical or similar results. GraphPad 8.0 software was used for data statistics and correlation analysis. Data represent biological replicates. Appropriate statistical tests were used for every figure. Mean ± SD is plotted in all figures. The comparison between 2 groups was analyzed with the Student *t* test. For data from 3 or more groups, statistical significance was determined based on ANOVA with Bonferroni correction. *P* < 0.05 is considered to be significant.

## RESULTS

### Aβ increased in livers and blood of infants with BA

**Figure 1.**
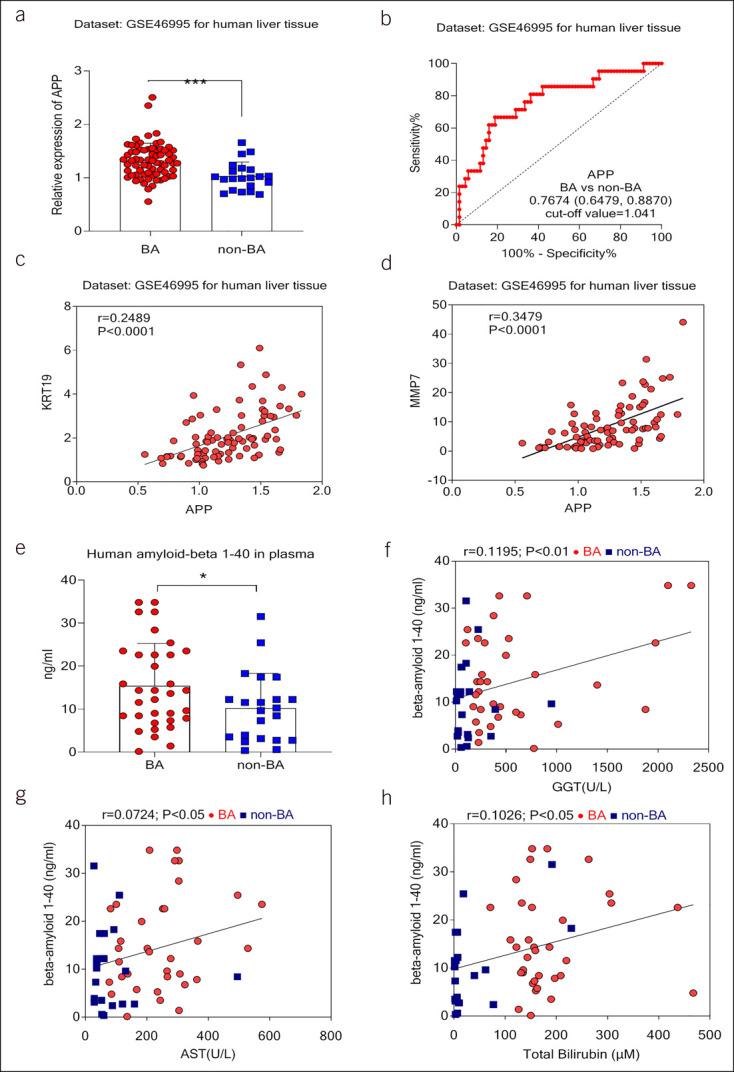
Beta-amyloid (Aβ) increased in blood and livers of patients with biliary atresia (BA). (**a**) Data for human amyloid beta (Aβ) precursor protein (APP) mRNA expression extracted from the gene expression omnibus (GEO) database, comparing whole extracts of liver tissue from human patients diagnosed with BA (n = 69) and non-BA cholestatic diseases (non-BA) (n = 21) (GSE46995). (**b**) The operating characteristic curve (ROC) for APP mRNA in BA vs non-BA. (**c**) The linear regression analysis showed that hepatic mRNA level of KRT19 was positively correlated with the expression of KRT19 in livers of patients with BA. (**d**) Hepatic mRNA level of APP was positively correlated with the expression of MMP7 in livers of patients with BA. (**e**) The contents of plasma Aβ 1–40 in 34 patients with BA and 22 patients without BA were determined. (**f**–**h**) The linear regression analysis showed that plasma Aβ 1–40 was positively correlated with gamma-glutamyl transferase (GGT), aspartate aminotransferase (AST), and total bilirubin (*P < 0.05; ***P < 0.001).

A data set was reanalyzed to assess the diagnostic and pathobiological potentials of Aβ for BA (17). The results suggested that Aβ precursor protein (APP) mRNA levels in BA liver tissues were higher in BA livers compared with that of non-BA livers (Figure 1a). To make clear whether hepatic APP levels play predictive roles for BA, the receiver operating characteristic curve (ROC) for APP was performed. The ROC for APP mRNA in BA vs non-BA was 0.7674 (95% confidence interval 0.6479–0.8870; *P* = 0.0002; Figure 1b), and the cutoff value of the ROC was 1.041. Hepatic mRNA levels of APP were positively correlated with matrix metallopeptidase-7 (*r* = 0.3479, *P* < 0.0001) mRNA level and keratin 19 (*r* = 0.2489, *P* < 0.0001) mRNA level (Figure 1c,d). With advances in BA diagnosis, including elevated blood levels of matrix metalloproteinase-7 (18–21), we hypothesized that Aβ in the blood may contribute to the diagnosis of BA. To test this hypothesis, we quantified plasma Aβ in 34 patients with BA and 22 patients without BA using an enzyme-linked immunosorbent assay. The concentrations of plasma Aβ were significantly elevated in patients with BA in relation to that of patients without BA (Figure 1e). We performed several correlation analyses of plasma liver injury progression with Aβ. The results showed the correlation plasma Aβ was significantly positively correlated with gamma-glutamyl transferase (*r* = 0.1195, *P* < 0.01), aspartate aminotransferase (*r* = 0.0724, *P* < 0.05), and T-bilirubin (*r* = 0.1026, *P* < 0.05) content (Figure 1f–h). In a Rhesus rotavirus A–infected mouse model (17), mouse APP mRNA level increased in the extrahepatic bile duct and gallbladder *en bloc* from the 3rd day after Ross River virus infection (see Supplementary Figure 1, http://links.lww.com/CTG/A881).

### Aβ accumulated around the central vein in livers of patients with BA

**Figure 2.**
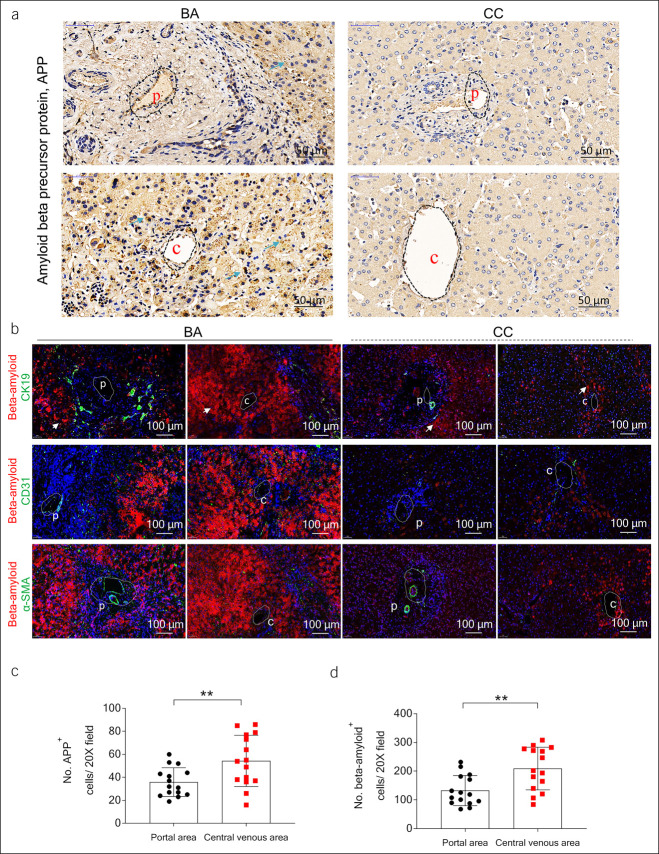
Beta-amyloid (Aβ) deposited around the central vein of patients with biliary atresia (BA). (**a**) Immunohistochemistry of amyloid beta (Aβ) precursor protein (APP) in the liver tissues of patients with BA and controls (benign liver tissues from choledochal cysts, CC, n = 5). (**b**) Immunofluorescent costained Aβ with CK19, PECAM-1/CD31, and α-smooth muscle actin in the liver tissues of patients with BA and controls. (**c**, **d**) Quantification of (**a**) and (**b**) of patients with BA (**P < 0.01).

To explore different geographical regions of the APP, immunohistochemistry was performed and showed the APP increasingly expressed in livers of BA compared with CC. APP was more likely to accumulate around the central vein (Figure 2a). In consistent with the previous findings (16), we showed that Aβ protein accumulated in the livers of patients with BA (Figure 2b). Aβ and its precursor protein deposition were often detected around the central vein (Figure 2c,d). Aβ was abundantly expressed in cells such as hepatocytes around the central vein of the liver of patients with BA (Figure 2a,b). In addition, compared with CC, Aβ protein accumulated and deposited in the biliary epithelial cells, CD31-positive endothelial cells, and α-smooth muscle actin–positive fibrotic niche in the livers of BA (see Supplementary Figure 2, http://links.lww.com/CTG/A881).

### Aβ inhibited growth of liver organoids

**Figure 3.**
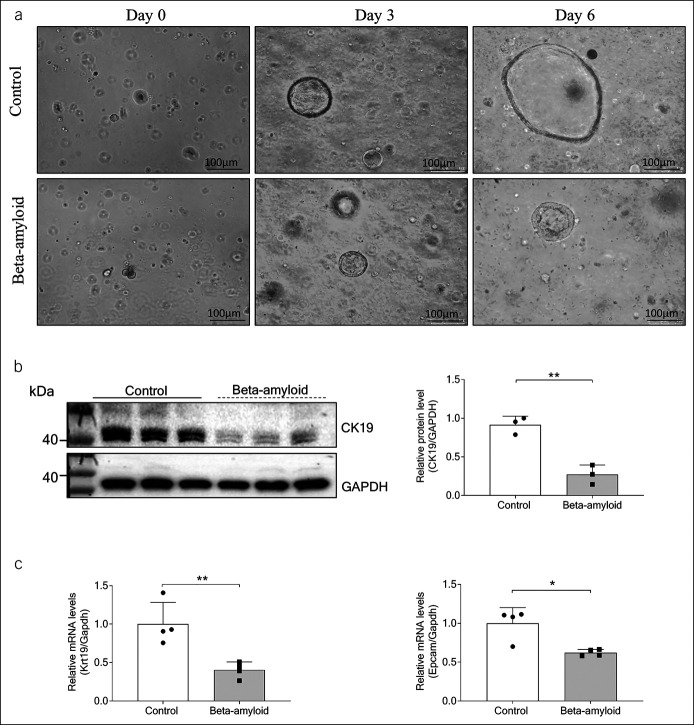
Beta-amyloid (Aβ) induced aberrant growth in liver organoids. (**a**) Representative bright field images of the liver organoids in day 0, day 3, and day 6. (**b**) Western blotting analysis for CK19 treated with BSA and 100-nM Aβ 1–42 and the quantifications. Representative images of the immune blottings are shown. (**c**) The Krt19, EpCAM gene levels were detected in organoids treated with BSA and 100-nM Aβ 1–42 by reverse transcription polymerase chain reaction assay. GAPDH was used as an internal control. Data were expressed as mean ± SD. BSA, bovine serum albumin; GAPDH, glyceraldehyde-3-phosphate dehydrogenase (*P < 0.05; **P < 0.01).

An organoid of murine liver organoids was established and treated with or without Aβ for 6 days (see Supplementary Figure 3A, http://links.lww.com/CTG/A881). By measuring the diameter of the organoids, it was found that organoids treated with Aβ had much smaller diameter and presented as thick-walled spheres (Figure 3a and see Supplementary Figure 3B, http://links.lww.com/CTG/A881). Multidrug resistance protein 1 encodes a transmembrane export pump in cholangiocytes and pumps compounds into the lumen of organoids, and this function can be blocked by verapamil. In attempt to test the organoid cell tight junctions, we added the fluorogenic substrate rhodamine 123 (R123) and verapamil to the culture medium of organoids. It was observed that R123 accumulated in the Aβ-treated organoid lumen, whereas control organoids showed less accumulation (see Supplementary Figure 3C, http://links.lww.com/CTG/A881). Western blotting showed that the organoids exhibited the characteristic of reduced cell differentiation and linked to decreased expression of biliary marker cytokeratin 19 (CK19) protein after administration of Aβ (Figure 3b). Consistently, organoids treated with Aβ showed downregulation of relative mRNA levels of cytokeratin 19, EpCAM, and HNF4a compared with the control group (Figure 3c and see Supplementary Figure 3D, http://links.lww.com/CTG/A881).

### Aβ impaired mitochondrial respiration in liver organoids

**Figure 4.**
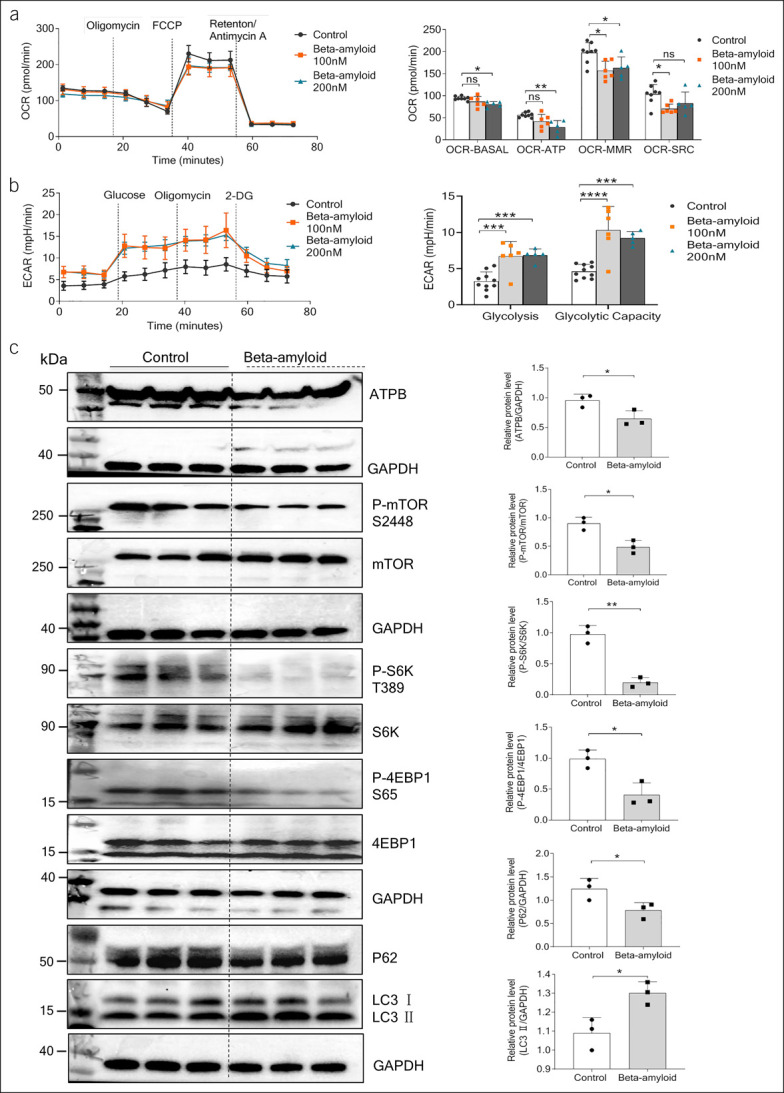
Beta-amyloid (Aβ) impaired mitochondrial respiration in liver organoids. (**a**) Oxygen consumption rate (OCR) of organoid-treated BSA and Aβ 1–42 (n = 5–10 experimental replicates). Basal respiration (OCR-BASAL), ATP production (OCR-ATP), maximal respiration (OCR-MMR), and spare respiratory capacity (OCR-SRC) were measured. (**b**) Extracellular acidification rate (ECAR) measurement and levels of glycolysis and maximal glycolytic capacity were recorded over time in organoid-treated BSA or Aβ 1–42. (**c**) Western blotting analysis for ATPB, p-mTOR, mTOR, p-S6K, S6K, P-4EBP1, 4EBP1, P62, and LC3 in cholangiocyte treated with BSA and 100-nM Aβ 1–42 and quantifications (*P < 0.05; **P < 0.01; ***P < 0.001; ****P < 0.0001). ATPB, ATP synthase, H+ transporting, mitochondrial F1 complex, beta polypeptide; BSA, bovine serum albumin; mTOR, mammalian target of rapamycin; ns, not significant.

To determine how Aβ affects organoid growth, energy metabolism was studied in detail. The real-time oxygen consumption rate (OCR) on sequential treatment with mitochondrial inhibitors in organoids was measured using a Seahorse XF96 Extracellular Flux Analyzer. Organoids treated with 100-nM Aβ exhibited significantly lower maximal respiration (OCR) and spare respiratory capacity (OCR) compared with controls. About 200-nM Aβ also altered mitochondrial energetic metabolism in organoids characterized by decreased baseline respiration (OCR-BASAL), ATP-linked respiration (OCR-ATP), and OCR maximal respiration (Figure 4a). To further characterize the glycolytic pathway, a marker of glycolysis, extracellular acidification rate was directly measured. By the time 10-mm glucose was added, extracellular acidification rate measurements revealed a significantly increased rate of glycolysis in Aβ-treated organoids compared with control group. The subsequent addition of the ATP synthase inhibitor oligomycin A revealed a significantly increased maximum glycolytic capacity in Aβ-treated organoid when compared with control group (Figure 4b). As shown in Figure 4b, glycolysis and glycolytic capacity were all substantially increased in Aβ-treated organoids compared with control group. Western blot analysis showed that administration of Aβ resulted in downregulation of ATPB protein expression (Figure 4c). The relative mRNA expression levels of central mitochondrial proteins, including ATP synthase, H^+^ transporting, mitochondrial F0 complex, subunit D (Atp5h), solute carrier family 25 member 3 (Slc25a3), calpain 1 (Capn1) and dynamin-like 120 kDa protein (Opa1), and uncoupling protein 2, were reduced evidently in response to Aβ treatment (see Supplementary Figure 4, http://links.lww.com/CTG/A881).

### Aβ suppressed mTOR signaling and increased autophagy

Western blot analyses revealed that phosphorylation of mTOR (S2448) was reduced in the presence of Aβ (Figure 4c). One of the major substrates of mTOR known is the p70 ribosomal protein S6 kinase (p70S6K) (22), a regulator of mRNA translation. mTOR phosphorylates and activates p70S6K at T389 to activate the ribosomal protein S6 through phosphorylation at S235/236 and S240/244 (23). Inhibition of ATP synthase results in reduced mTOR signaling in multiple organisms (24). We found that 4-hour administration of 100-nM Aβ decreased the phosphorylation of P70S6K (Figure 4c). mTOR-directed eukaryotic translation initiation factor 4E-binding protein 1 (4EBP1) phosphorylation promotes cap-dependent translation (25). Aβ treatment also decreased the phosphorylation of 4EBP1 (Figure 4c). We were next to detect autophagy-related proteins P62 and LC3. It showed that the expression of P62 protein decreased and the expression of LC3 protein increased in the Aβ-treated group (Figure 4c).

### Aβ suppressed liver development in zebrafish

**Figure 5.**
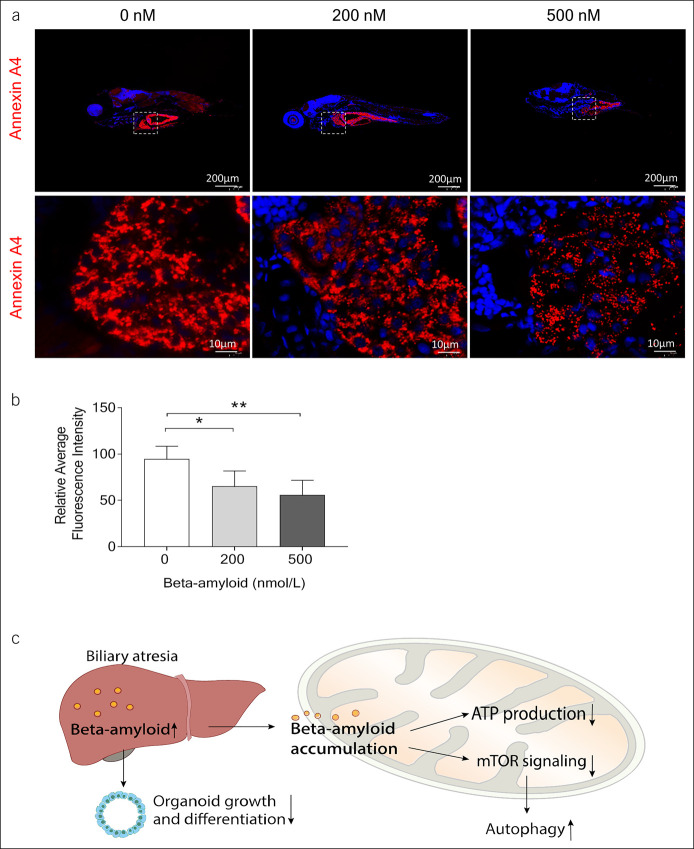
Beta-amyloid (Aβ) suppressed liver development in zebrafish. (**a**) Representative image of immunofluorescent-stained annexin A4 (n = 5). (**b**) Quantification of (**a**). (**c**) Proposed mechanism involving Aβ in the pathogenesis of biliary atresia (*P < 0.05; **P < 0.01).

Hypoplastic intrahepatic ducts with short ductular projection mean limited liver development (26). In this article, zebrafish larvae (5 dpf) were exposed to various concentrations of Aβ (200–500 nM) for 24 hours. The liver development was then analyzed by using immunofluorescence staining with a monoclonal antibody against annexin A4, a marker of biliary tract. It showed that Aβ-treated larvae had lower relative expression annexin A4 and smaller livers (Figure 5a,b).

## DISCUSSION

BA is a severe cholestatic liver disease in neonates and often not diagnosed at the right time (5). In this study, we found Aβ expression increased in the plasma and livers of infants with BA. Liver organoids treated with Aβ had abnormal morphology and impaired growth. We also observed that Aβ suppressed the mitochondrial respiration in liver organoids by decreasing mitochondrial respiration and altering mTOR signaling. Aβ exposure caused liver abnormal development in zebrafish larvae (Figure 5c).

Aβ has traditionally been characterized as a nonfunctional catabolic byproduct. It plays an antibacterial physiological function in the body at a lower concentration (27). Aβ dyshomeostasis caused by various factors may result in Aβ accumulation and aggregation (28). We found here a significant increase in BA plasma Aβ levels compared with controls, which suggested values of plasma Aβ have discriminatory features for BA, which is confirmed as a diagnostic characteristic for Aβ in BA that was proposed by a previous study (16). Furthermore, the current study showed the relative mRNA expression level of Aβ precursor protein APP was increased in patients with BA and a significant positive correlation between matrix metallopeptidase-7, a potential diagnostic marker for BA. In the livers of BA, the Aβ protein was found to be mainly deposited around the central vein area and suggested Aβ may be involved in the liver injury and metabolism of BA. Indeed, we indicated that administration of Aβ induced liver organoids presenting growth restriction as well as impaired morphology and reduced organoid differentiation. Previous studies reported that BA-like cholangiocyte organoids presented cholangiocyte monolayer damage and increased permeability (29). Our study here confirmed Aβ created weaker cell tight junctions, which suggested Aβ-caused biliary ducts lost the ability of preventing the toxicity of secreted bile acids (30). Little is known, however, about how Aβ modifies the organoids and cell growth. Our findings revealed that Aβ increased glycolysis and decreased ATP synthesis. Previous studies have found that Aβ drives up neuronal and microglia aerobic glycolysis (31,32). Some studies have also been indicated that Aβ caused mitochondrial Ca^2+^ overload and mitochondrial dysfunction by destabilizing intracellular Ca^2+^ homeostasis (33–36). It is thus hypothesized that Aβ shifted the mitochondrial respiration to glycolysis and leaded to reduce energy metabolism in liver cells. Dephosphorylation of mTOR, p70S6K, and 4EBP1 (the immediate downstream of mTOR) showed Aβ also regulated oxidative phosphorylation through inhibition of ATP synthase and mTOR signaling downstream. Metabolic characterization of intact cells reveals intracellular Aβ but not its precursor protein to reduce mitochondrial respiration (37). Altered mTOR signaling levels correlate with autophagy. Cellular energy metabolism is reduced after Aβ administration, and energy deprivation can rapidly activate autophagy (38,39). In this study, we also observed that autophagy increased in response to Aβ treatment and suggested possible mechanisms mediated by Aβ in the pathogenesis in the liver injury and regeneration. Our studies also confirmed that Aβ deposits impaired liver development *in vivo*, evidenced by Aβ-giving zebrafishes showed lower intrahepatic bile duct density.

In conclusion, increased Aβ in organoids and zebrafishes attenuated mitochondrial respiration and mTOR signaling, activated autophagy, and thus affected liver regeneration. This study may help enrich the treatment of BA by improving Aβ metabolism.

## CONFLICTS OF INTEREST

**Guarantors of the article:** Wei Cai, MD, PhD, and Yongtao Xiao, PhD.

**Specific author contributions:** Y.X., W.C., and Y.W.: developed study concept and design, acquisition of data, analysis, and interpretation of data. Y.X.: wrote the manuscript. X.T., W.W., B. W., J.D., and Y.L.: performed and analyzed most of the experiments. W.C., Y.Z., and Y.W.: reviewed and revised the manuscript. All the authors approved this version of the manuscript to be published.

**Financial support:** This study was supported by the National Natural Science Foundation of China (82270537 and 81974058) and Shanghai Key Laboratory of Pediatric Gastroenterology and Nutrition (17DZ2272000), Foundation of Science and Technology Commission of Shanghai Municipality (19495810500), and Foundation of Shanghai Municipal Health Commission (shslczdzk05702).

**Potential competing interests:** None to report.

**Data availability statement:** The data that support the findings of this study are available from the corresponding authors (X.Y. and C.W.) on reasonable request.

**Study Highlights**

**WHAT IS KNOWN**

✓ Beta-amyloid (Aβ) significantly increased in livers of infants with biliary atresia (BA) and deposited around the central vein.

**WHAT IS NEW HERE**

✓ Aβ elevated significantly in BA plasma and positively correlated with liver injury progression.
✓ Aβ impaired mitochondrial respiration and increased glycolysis in organoids.
✓ The mammalian target of rapamycin signaling was suppressed by Aβ administration *in vitro*.

## Supplementary Material
