# Supplementary material for: Beta-amyloid Deposition in Biliary Atresia Reduces Liver Regeneration by Inhibiting Energy Metabolism and Mammalian Target of Rapamycin Signaling
Source: Clin Transl Gastroenterol. 2022 Sep 22;13(11):e00536. doi: 10.14309/ctg.0000000000000536 (PMC10476755; doi:10.14309/ctg.0000000000000536)

**Beta-amyloid Deposit in Biliary Atresia Reduces liver regeneration via Inhibiting Energy Metabolism and mTOR signaling**

**Content**

Supplementary Materials and Methods, Page 2 - 9

Supplementary Tables and Figures

Table S1，Page 11 -12

Table S2，Page 13

Table S3，Page 14 -15

Table S4，Page 16

Figure S1, Page 17

Figure S2, Page 18

Figure S3, Page 19

Figure S4, Page 20

Original and uncropped western-blot bands, Page 21 - 23

**Supplementary Materials and Methods**

**Real-time quantitative polymerase chain reaction (qRT-PCR)**

After administration of 100 nM BSA or 100 nM Aβ 1-42, the total RNA was extracted from human liver samples using the RNeasy kit according to manufacturing procedures. RNA from organoids was extracted using RNAprep Pure Micro Kit. Briefly, add 500 µL of cold PBS. Gently pipette up and down several times to resuspend the organoids in the well. Transfer the mixture to 1.5 mL tube. Centrifuge the mixture at 600 g for 5 min at 4 °C. Total RNA was then extracted according to the reagent manufacturer's instructions. RNA volumes were determined using nanocrystal spectroscopy. Hifair® III 1st Strand cDNA Synthesis SuperMix was employed for reverse transcription. Then, ViiA 7 Real-Time PCR system (Applied Biosystems, Foster City, CA) and Hieff® qPCR SYBR Green Master Mix were used for real-time PCR reaction. PCR reaction was incubated at 95°C for 10 min, 95°C for 15 s, and 60°C for 1 min in 384 well plates, with 40 cycles. The reagents used in this study were listed in Table S2. All samples were 3 replicates and data were normalized to endogenous control Gapdh. Relative RNA expression was calculated using ΔΔ Ct method. Primers were listed in Table S3.

**Immunohistochemistry (IHC)**

Paraffin-embedded sections were cut at 3 μm, dried at 70°C for 30 minutes and then dewaxed. Antigen retrieval was performed in a repair box filled with citric acid (pH 6.0) antigen retrieval buffer for 23 minutes. Endogenous peroxidase was blocked with 3% H_2_O_2_ for 25 minutes at room temperature (RT) and then sections were incubated with 3% BSA for 30 minutes. The primary antibody of amyloid beta were incubated in wet chamber overnight at 4 ℃ and secondary antibody was incubated for 50 minutes at RT. The sections were successively stained with DAB color developing solution. After counterstaining with hematoxylin, the sections were observed under a microscope. The slices were finally dehydration with xylol and ascending concentrations of ethanol and mounted with SweSuper Clean BioMount Medium. Images were photographed using a Grundium Ocus Device (MGU-00001, Grundium Ltd, Tampere, Finland) and analyzed by the ImageJ software (NIH, Bethesda, MD, USA).

**Immunofluorescence (IF)**All the liver tissues were immediately fixed in 4% paraformaldehyde for 24 h and went through dehydration, clearing and paraffin embedding. Sections were mounted on positively charged slides after cutting at 4 μm thick, baked at 65 °C for 1 h and then stored at room temperature for later use. Immunofluorescence was performed using the chromogen diaminobenzidine as we described previously (20). Briefly, the slides were incubated with xylol and descending concentrations of ethanol. Endogenous peroxidases were blocked by using 3% H_2_O_2_ for 10 min at RT. After antigen retrieval, blocking was performed using 5% bovine serum albumin for 30 min at RT. The antibodies were applied at their optimal concentration overnight in a wet chamber for overnight at 4 °C. The slides were rinsed in phosphate-buffered saline (PBS) and incubated with the appropriate secondary antibody for 1 h at RT. Antibody binding was visualized using a liquid diaminobenzidine Substrate Chromogen System. The slides were rinsed in PBS and counterstained with hematoxylin. I Immunofluorescence images analysis was used software Case Viewer 4 fields/sample (20 different fields were analyzed from 5 different samples from 5 different patients). The primary antibodies performed in this study were listed in Table S4.

**Enzyme Linked Immunosorbent Assay (ELISA)**

Aβ1-40 accounts for about 90% of the total secreted Aβ and is the main soluble Aβ in biological fluids(1). To determine the level of Aβ in plasma, Aβ 1-40 in plasma was examined with a Amyloid beta 1-40 Human ELISA Kit (ab193692, Abcam). ELISA was performed according to the manufacturer's instructions. Briefly, prepare all reagents, samples and standards as instructed in the beginning. A 96 well plate was used, 100 μL of each standard and sample was added into appropriate wells. The immobilized antibody captured amyloid beta 1-40 present in the samples and standards. Biotinylated anti-Human amyloid beta 1-40 antibody was added follow washing wells. After subsequent washing to remove unbound biotinylated antibody, HRP-conjugated streptavidin was pipetted into the wells, incubate for 45 minutes. Add TMB substrate solution and the color in the wells was proportional to the amount of amyloid beta 1-40 bound in each well. Finally add Stop Solution and read at 450 nm.

**Magnetic Associated Cell Sorting (MACS)**

Tissue dissociation was carried out as previous described(2). Briefly, the mice were anesthetized and dripped with Hank’s balanced salt solution (HBSS) without Ca^2+^, Mg^2+^ through portal vein at 25-30 mL/min, while the inferior vena cava had been cut. HBSS with Ca^2+^, Mg^2+^ containing 0.05% collagenase Ⅱ was then perfused until white tree structures are visible. Resect the tree structures, gently shake it in PBS to remove other parenchymal cells. Cut up the tree structure, suspend the fragments in 10 mL digestion medium (2 mg/ml collagenase Ⅳ, 0.01 mg/ml DNase Ⅰ), shake the tubes at 37 °C for 30 min. Filtrate the digested tissue with a 40 μm cell strainer. Centrifuge the tube at 800 × g for 10 min at 4 °C to get single cells. Following tissue dissociation to single cells, cholangiocytes were isolated with Magnetic Associated Cell Sorting (MACS) using the CD326 (EpCAM) MicroBeads according to the manufacturer’s instructions. Briefly, every 10^7^ total cells were re-suspended in 90 µL buffer，added 10 μL CD326 (EpCAM) MicroBeads and incubate for 15 minutes at 4° C. Pass the mixture through a LS column. Then the cells were washed down with running buffer. The resulting cells were counted, centrifuged at 444 g for 5 minutes, resuspended to a concentration of 1000 cells/ μL and stored on ice.

**Culture of organoids**

The cell pellet (approximately 1x10^5^ cells) was first mixed with 15 µl cold serum-free medium, then mixed with 40 µl of Matrigel and seeded per well of a prewarmed (37^o^ C) 24-well plate. The mixture was added to the bottom of well to form a dome, plates were firstly incubated at 37^o^ C for 5-10 min; then incubated upside-down (matrigel up) at 37^o^ C for 5 min again to allow the cells evenly distribute within the matrigel as well as to avoid accumulation of cells towards the bottom. After Matrigel had solidified, 500 µl organoid medium was added to each well. Organoid medium was based on Advanced DMEM/F12 and a conditioned medium from L-WRN cells (3), supplemented with Penicillin/Streptomycin, 25 mM HEPES, 1% N2, 1% B27, 50 ng/ml EGF, 100 ng/ml FGF10. First three days of culture, we added 10 µM ROCK inhibitor Y-27632. Medium was changed once every two days. Passage zero (P0) primary organoids were used for all the experiments.

**Passaging and freezing of organoids**

The organoids are usually passed on 7-9 day when organoids grew beyond the size limit. For passaging, the matrix and organoids are pipetted up and down into small pieces in 500 µl ice-cold Advanced DMEM/F12 medium. The disrupted organoids were next transferred to 15 ml tubes, add 10 ml of Advanced DMEM/F12 medium and place it on ice for 10-15min to dissolve the matrigel. The samples were centrifuged at 4ºC at 300 g for 10 min. The supernatant was aspirated, and the cell pellets were resuspended in 50-60 µl of matrigel. About 500 µl of prewarmed medium was then added per well after solidification of matrigel. The medium was changed every alternate day for 1 to 2 weeks.

**Beta-amyloid administration on organoids**

Amyloid beta protein fragment 1-42 was dissolved to 1 mM in DMSO and added to single cells produced by organoid digestion at a final concentration of 100 nM. Single cells were obtained as following described: The organoid medium along with matrix gel containing organoids was aspirated from each well, transferred to a 15 ml tube, 1-2 ml cold Advanced DMEM/F12 was added and the mixture was incubated on ice for 10 min to dissolve matrix gel before centrifugation (300 g, 5 min). The supernatant was aspirated until the organoid pellet and a layer of matrix gel remained. 1 ml of 5X TrypLE Express was added, mixed well and incubated at 37°C for 5 min. 1 ml of FBS was added to the mixture, which was pipetted up and down for 40-50 times with a small circumference opening glass pipette (diameter 0.3 - 0.5 mm) to efficiently dissociate organoids into single cells. 5-10 ml of cold Advanced DMEM/F12 medium was added, and the suspension was passed through a 40 µm strainer before centrifugation (300 g; 5 min) at 4^o^ C. The supernatant was aspirated until only the pellet remained and the cells were counted following addition of 200 µl organoid medium. After dilution, about 1000 cells were divided into different tubes and centrifuged (300 g, 5 min) at 4^o^ C, to remove the medium. Four replicates each for control (DMSO with 100 nM BSA) and treatments were analyzed. 500 µl of organoid medium containing 100 nM BSA dissolved in DMSO for control and beta-amyloid protein fragment (100 nM final concentration) for each treatment was prepared. The cell pellets were incubated (10 min) with 10 µl of prepared medium with appropriate compounds. 50-60 µl of matrigel was added, and the rest of the corresponding medium with compounds (totally 500 µl) was added per well after solidification of matrigel. The medium was changed with corresponding concentrations of beta-amyloid protein fragment 42 (100 nM).

**Rhodamine transport assay**

Organoids were incubated with 100 µM Rhodamine 123 in organoid culture medium at 37°C for 5 minutes and then washed three times with the culture medium. Organoids were then incubated in culture medium with or without the addition of Verapamil (10 µM) at 37°C for 40 minutes. Organoids were imaged using Laica Software.

**Oxygen consumption rates measurement (OCR).**

About 10 thousand cells were planted in an XF96 well plate coated with a 1:10 dilution of Matrigel in PBS and allowed to sit at room temperature for 1 h. Organoids were evenly seeded in each well with the organoid culture medium. The following day, growth media was changed to bicarbonate-free assay media with BSA or Aβ 1-42 and incubated at 37 °C for 1 h in a CO_2_-free incubator. Organoids were run on an XF96 Analyzer for a Mito Stress Test in standard assay medium (DMEM, 25 mM glucose, 2 mM pyruvate, 2 mM glutamine, pH 7.4) using the manufacturer’s protocol and tested drug concentrations (1 µM oligomycin, 1.2 µM FCCP, 0.5 µM rotenone, and 0.5 µM antimycin A).

**Extracellular acidification rates measurement (ECAR).**

About 10 thousand cells were planted in an XF96 well plate, which was coated with a 1:10 dilution of Matrigel in PBS and allowed to sit at room temperature for 1 h. Organoids were evenly seeded in each well with the organoid culture medium. The following day, growth media was changed to bicarbonate-free assay media with BSA or Aβ 1-42 and incubated at 37 °C for 1 h in a CO_2_-free incubator. Extracellular acidification rate (ECAR) was measured using an XF96 Extracellular Flux Analyzer under basal conditions and following addition of 10 mM glucose, 1 μM Oligomycin, and 50 mM the glucose analog, 2-deoxyglucose, 2DG, according to the manufacturer’s protocol.

**Western blotting**

After administration of vehicle of 100 nM Aβ 1-42. Briefly, about 1 X 10^5^ cells was homogenized in 200 μL RIPA buffer supplemented with a protease inhibitor cocktail. BCA reagent was used to determine the protein concentration. The equal amounts of protein were separated on NuPAGE 10% Bis-Tris gels and transferred onto polyvinylidene difluoride (PVDF) membranes. After blocking in 5% nonfat milk at room temperature for 60 min, membranes were incubated with the primary antibodies overnight at 4 °C. The membranes were washed three times for 30 min with TBST (containing 0.1% Tween-20), and then incubated with secondary antibodies at room temperature for 10 min. After final washes with TBST, the signals were detected using ECL chemiluminescence reagent Kit. The primary antibodies performed in this study were listed in Table S4.

**Zebrafish bioassay and analyses**

Zebrafish larvae (5dpf) were exposed to Aβ 1-42 dissolved in embryo medium at a concentration of 200-500 nmol/L. Every 15 larvae were placed in a well of a 24-well plate for 24 hours. Change the medium containing each compound every 12 hours. All tests were repeated three times with at least 30 treated larvae and the same number of control larvae. After treatment, the larvae were fixed with 4% paraformaldehyde and treated with Annexin A4 antibody for immunofluorescent staining. Relative average fluorescence Intensity was analyzed with Image J software (NIH Image, Bethesda, MD).

**Supplemental References**

1. Zou K, Kim D, Kakio A, et al. Amyloid beta-protein (Abeta)1-40 protects neurons from damage induced by Abeta1-42 in culture and in rat brain. J Neurochem 2003;87:609-619.

2. Katsuda T, Ochiya T, Sakai Y. Generation of Hepatic Organoids with Biliary Structures. Methods Mol Biol 2019;1905:175-185.

3. Shiota J, Zaki NHM, Merchant JL, et al. Generation of Organoids from Mouse Extrahepatic Bile Ducts. J Vis Exp 2019.

**Table S1 The information of patients with biliary atresia and control subjects**

| **NO** | **Gender** | **Age**  **（d）** | **ALT (U/L)** | **AST (U/L)** | **γGT (U/L)** | **T-Bilirubin (μM)** | **D-Bilirubin (μM)** | **Primary disease** |
| --- | --- | --- | --- | --- | --- | --- | --- | --- |
| BA 1 | F | 43 | 126 | 202 | 215 | 156.4 | 114 | BA |
| BA 2 | F | 11 | 115 | 278 | 230 | 143.5 | 69 | BA |
| BA 3 | M | 40 | 193.2 | 108.2 | 315 | 122 | 90 | BA |
| BA 4 | F | 65 | 172 | 252 | 1975 | 436.9 | 312.7 | BA |
| BA 5 | M | 29 | 40 | 85 | 348 | 467.4 | 265.5 | BA |
| BA 6 | M | 46 | 142 | 244 | 235 | 189.8 | 151.4 | BA |
| BA 7 | F | 61 | 162 | 209 | 2099 | 182.4 | 134.5 | BA |
| BA 8 | F | 72 | 109 | 115 | 263 | 110.3 | 58.9 | BA |
| BA 9 | M | 65 | 255 | 366 | 788 | 145.8 | 110.9 | BA |
| BA 10 | M | 56 | 132 | 208 | 1400 | 158.9 | 87.3 | BA |
| BA 11 | F | 72 | 255 | 305 | 434 | 263.2 | 185.5 | BA |
| BA 12 | F | 61 | 125 | 324 | 423 | 154 | 112.9 | BA |
| BA 13 | F | 25 | 40 | 78 | 645 | 157.4 | 77.4 | BA |
| BA 14 | M | 82 | 208 | 308 | 443 | 134.7 | 107.5 | BA |
| BA 15 | M | 39 | 91 | 136 | 775 | 150.1 | 85.8 | BA |
| BA 16 | F | 53 | 122 | 266 | 375 | 135.1 | 109.8 | BA |
| BA 17 | F | 74 | 264 | 363 | 598 | 196.8 | 99.7 | BA |
| BA 18 | F | 38 | 44 | 82 | 289 | 71.3 | 51.4 | BA |
| BA 19 | F | 59 | 505 | 529 | 247 | 186.5 | 145 | BA |
| BA 20 | M | 28 | 136.5 | 257.7 | 104 | 152.5 | 73.94 | BA |
| BA 21 | M | 75 | 223 | 267 | 251 | 166 | 134.1 | BA |
| BA 22 | F | 70 | 156 | 235 | 1014 | 159.9 | 105.9 | BA |
| BA 23 | M | 65 | 67.5 | 304.9 | 229 | 126.4 | 46.08 | BA |
| BA 24 | F | 58 | 377.4 | 574.8 | 223 | 132.5 | 58.21 | BA |
| BA 25 | F | 14 | 31.1 | 100.8 | 527 | 307.4 | 80.25 | BA |
| BA 26 | F | 77 | 315 | 304 | 377 | 121.6 | 95.5 | BA |
| BA 27 | F | 80 | 177 | 298 | 2327 | 152.7 | 110.7 | BA |
| BA 28 | M | 81 | 141.1 | 183.1 | 499 | 212.5 | 110.65 | BA |
| BA 29 | F | 76 | 296 | 496 | 120 | 303.7 | 182.6 | BA |
| BA 30 | M | 30 | 59.9 | 109.7 | 201 | 219.7 | 60.09 | BA |
| BA 31 | M | 60 | 77 | 119 | 1877 | 208.7 | 159.7 | BA |
| BA 32 | F | 60 | 124 | 291 | 707 | 149.2 | 78.5 | BA |
| BA 33 | M | 60 | 151 | 167 | 203 | 162.7 | 81.2 | BA |
| BA 34 | F | 60 | 107 | 138 | 177 | 130.8 | 54.7 | BA |
| non-  BA 1 | M | 93 | 12 | 28 | 106 | 191.6 | 0 | CC |
| non-  BA 2 | M | 300 | 32 | 37 | 11 | 1.5 | ＜1 | DH |
| non-  BA 3 | M | 180 | 58 | 61 | 139 | 7.1 | 3 | CC |
| non-  BA 4 | M | 60 | 15 | 37 | 52 | 7.3 | 1 | CC |
| non-  BA 5 | M | 210 | 215.7 | 131.1 | 948 | 61.9 | 10.18 | CC |
| non-  BA 6 | M | 360 | 31.2 | 53 | 29 | 2.2 | 0 | CC |
| non-  BA 7 | M | 360 | 28.1 | 47.1 | 59 | 6.6 | 0 | CC |
| non-  BA 8 | M | 720 | 13 | 37 | 48 | 3.9 | 1.2 | CC |
| non-  BA 9 | F | 420 | 334 | 495 | 394 | 40.1 | 28.6 | Cholangi-  tis |
| non-  BA 10 | F | 12 | 59.5 | 55.6 | 14 | 7.3 | 0 | DH |
| non-  BA 11 | F | 43 | 27.9 | 93.1 | 106 | 229.4 | 0 | CC |
| non-  BA 12 | M | 90 | 34 | 38 | 20 | 1.5 | 18 | ASD |
| non-  BA 13 | M | 90 | 112 | 111 | 225 | 18.5 | 4.6 | CC |
| non-  BA 14 | F | 60 | 176 | 160 | 352 | 10.5 | 4.7 | CC |
| non-  BA 15 | F | 90 | 126 | 121 | 21 | 9.2 | 11.5 | CC |
| non-  BA 16 | F | 150 | 85 | 29 | 121 | 3 | 12.4 | CC |
| non-  BA 17 | F | 60 | 85 | 29 | 26 | 6.2 | 5.4 | ASD |
| non-  BA 18 | M | 180 | 81 | 34 | 66 | 2.4 | 5 | ASD |
| non-  BA 19 | M | 90 | 58 | 62 | 64 | 3.1 | 9.3 | CC |
| non-  BA 20 | F | 42 | 67 | 88 | 127 | 77 | 6.7 | DH |
| non-  BA 21 | M | 210 | 38 | 54 | 114 | 6.6 | 5 | CC |
| non-  BA 22 | M | 120 | 40 | 60 | 57 | 3.5 | 1.9 | CC |

ALT, alanine aminotransferase (9-52 U/L); AST, aspartate aminotransferase (14-36 U/L); AKP, (38-126 U/L); γGT γ-glutamyltranspeptidase (12-43 U/L); T-Bilirubin, total bilirubin (3.42-20.52 μM); D-Bilirubin, direct bilirubin (0-6.8μM); CC, choledochal cysts; DH, diaphragmatic hernia; ASD, atrial septal defect

**Table S2. Reagents and Source**

| **Reagent** | **Catalog#** | **Source** | |
| --- | --- | --- | --- |
| Human Amyloid beta 1-40 ELISA Kit | ab193692 | | Abcam |
| HBSS (Ca2+/Mg2+) | G4204-500mL | | Servicebio |
| HBSS (without Ca2+/Mg2+) | G4203-500mL | | Servicebio |
| CollagenaseⅡ | G5029 | | Servicebio |
| Collagenase Ⅳ | G5027 | | Servicebio |
| DNase I | G3342 | | Servicebio |
| CD326 (EpCAM) MicroBeads | 130-105-958 | | Miltenyi Biotec |
| LS column | 130-042-401 | | Miltenyi Biotec |
| Matrigel | 356237 | | Corning Biocoat |
| Advanced DMEM/F12 | G4612-500mL | | Servicebio |
| Penicillin/Streptomycin | 15140-122 | | Life Technologies |
| HEPES | 15630-080 | | Life Technologies |
| B27 | 17504-044 | | Gibco |
| N2 | 17502-048 | | Gibco |
| EGF | PMG8041 | | Invitrogen |
| FGF10 | 100-26-25UG | | Pepro tech |
| Amyloid beta Protein Fragment 1-42 | A9810 | | Sigma-Aldrich |
| L-WRN cells | CRL-3276 | | ATCC |
| RNAprep Pure Micro Kit | DP420 | | TIANGEN |
| Hifair® II 1st Strand cDNA Synthesis SuperMix | 11120ES | | Yeasen |
| Hieff® qPCR SYBR Green Master Mix | 11201ES | | Yeasen |
| Nuclear and Cytoplasmic Protein Extraction Kit | P0027 | | Beyotime |
| Enhanced BCA Protein Assay Kit | P0009 | | Beyotime |
| XF96 cell culture microplates | 101104-004 | | Seahorse Biosciences |
| XF assay medium | 102365-100 | | Seahorse Biosciences |
| XF DMEM medium | 103575-100 | | Seahorse Biosciences |
| Seahorse XF Glycolysis Stress Test Kit | 103020-100 | | Seahorse Biosciences |
| Seahorse XF Cell Mito Stress Test Kit | 103015-100 | | Seahorse Biosciences |

**Table S3 The primer sequences for qRT-PCR**

| **Genes** |  | **Sequence (5’-3’)** | **Product length (bp)** | **NCBI Gene ID** |
| --- | --- | --- | --- | --- |
| Gapdh | Forward | CCCTTAAGAGGGATGCTGCC | 124 | NM_001289726.1 |
|  | Reverse | TACGGCCAAATCCGTTCACA |  |  |
| Epcam | Forward | AACACAAGACGACGTGGACA | 113 | NM_008532.2 |
|  | Reverse | GCTCTCCGTTCACTCTCAGG |  |  |
| Opa1 | Forward | AGGCCCTTCTCTTGTTAGGT | 99 | NM_001199177.2 |
|  | Reverse | CTTTGTCTGACACCTTCCTGT |  |  |
| Capn1 | Forward | GGGGCTACCGTTTGTCTAGC | 95 | NM_001110504.1 |
|  | Reverse | CTCTGTCATCCTCTGGTGGC |  |  |
| Slc25a3 | Forward | AAGGTATTCACCCCAGGAAAAA | 83 | NM_133668.4 |
|  | Reverse | GGAGTTTCTTAAGTGAGGGGACA |  |  |
| Ucp2 | Forward | GGAAAATCGAGGGGATCGGG | 119 | NM_011671.5 |
|  | Reverse | GGAGTTCTGGAGGCTGCTTT |  |  |
| Krt19 | Forward | AAAACACTGAACCCTGATTCTTG | 94 | NM_001313963.1 |
|  | Reverse | TCTGAAGTCATCTGCAGCCA |  |  |
| Atp5h | Forward | GGGGGTCGGTGAAGTATCC | 87 | NM_027862.1 |
|  | Reverse | GGGGCATGACCTCCACAAAA |  |  |
| Hnf4a | Forward | CCCTTGGTCATGGTCAGTGT | 71 | NM_001312906.1 |
|  | Reverse | GACCCTGTGAGGGCATAAGG |  |  |

**Table S4 Antibody information**

| Antibody | Source | Catalog# | Application/dilution |
| --- | --- | --- | --- |
| beta Amyloid | abcam | ab11132 | IF (1:100) |
| Amyloid beta precursor protein | Servicebio | GB11307 | IF (1:500) |
| CK19 | Servicebio | GB11197 | IF/WB (1:500) |
| CD31 | Servicebio | GB11063-1 | IF (1:100) |
| Alpha-SMA | Servicebio | GB111364 | IF (1:500) |
| Annexin A4 | Servicebio | GB111147 | IF (1:500) |
| ATPB | PROTEINTECH | 17247-1-AP | WB (1:1000) |
| 4E-BP1 | Cell Signaling Technology | 9452P | WB (1:1000) |
| Phospho-4E-BP1 (Ser65) | Cell Signaling Technology | 9451P | WB (1:1000) |
| p70(S6K) | PROTEINTECH | 14485-1-AP | WB (1:1000) |
| Phospho-p70 S6K (Thr389) | PROTEINTECH | 28735-1-AP | WB (1:1000) |
| GAPDH | Affinity | AF7021 | WB (1:1500) |
| Phospho-mTOR(Ser2448) | Cell Signaling Technology | 2976P | WB (1:1000) |
| mTOR | Cell Signaling Technology | 2983P | WB (1:1000) |
| P62 | PROTEINTECH | 18420-1-AP | WB (1:1000) |
| LC3 | Cell Signaling Technology | 12741 | WB (1:1000) |

**Supplementary Figures**

**Figure S1. Beta-amyloid (Aβ) increased in Rhesus rotavirus A (RRA) infected mice.** Data for mouse beta-amyloid precursor protein (App) mRNA expression extracted from the GEO microarray database, comparing whole extracts of bile duct and gallbladder tissue from RRA infected mice (n = 3 animals per RRV-treated group or for saline group) at the day of 3, 7 and 14 (GSE46995).

**Figure S2.** **Aβ deposited in the liver of BA patients.**

Immunofluorescent co-stained beta-amyloid with CK19, PECAM-1/CD31 and α-SMA in the liver tissues of patients with BA and controls (benign liver tissues from choledochal cysts (CC)).

**
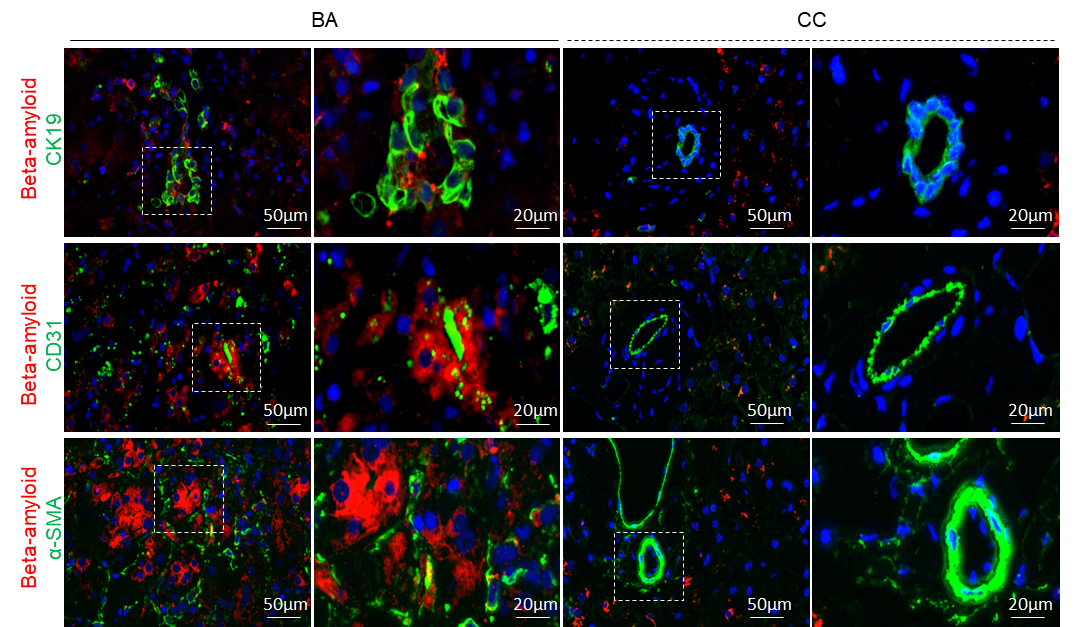
**

**Figure S3. Aβ induced aberrant growth and permeability in liver organoids.**

(A) Schematic pictures of the establishment of liver organoids from intrahepatic bile ducts. (B) Diameter (organoid size) of P0 (passage zero) primary organoids given BSA and 100 nM Aβ 1-42. (C) Representative images showing that verapamil inhibited the localization of the MDR1 fluorogenic substrate rhodamine 123 (R 123) within the lumen of Control organoids, but didn’t inhibit luminal R 123 localization in Aβ 1-42 treated organoids. (D) The Hnf4a gene levels were detected in organoids treated with BSA and 100 nM Aβ 1-42 by RT-PCR assay. GAPDH was used as an internal control. Data were expressed as mean ± SD. GAPDH, glyceraldehyde-3-phosphate dehydrogenase. *P < 0.05

**
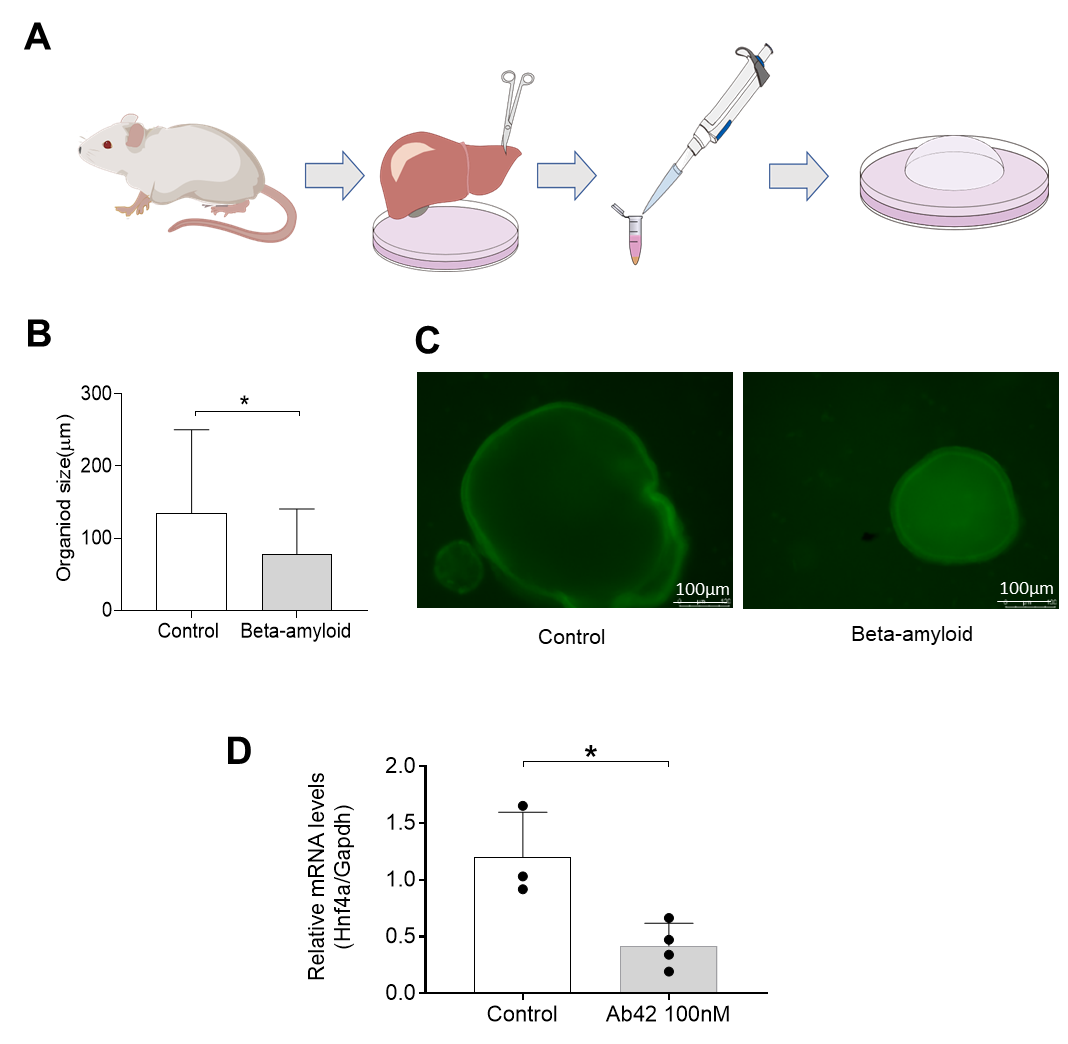
**

**Figure S4. Aβ suppressed energy metabolism in liver organoids.** The Atp5h, Slc25a3, Capn1, Opa1, and Ucp2 gene levels were detected in organoids treated with BSA and Aβ 1-42 by RT-PCR assay. GAPDH was used as an internal control. Data were expressed as mean ± SD. GAPDH, glyceraldehyde-3-phosphate dehydrogenase. *P < 0.05; ns, not significant.

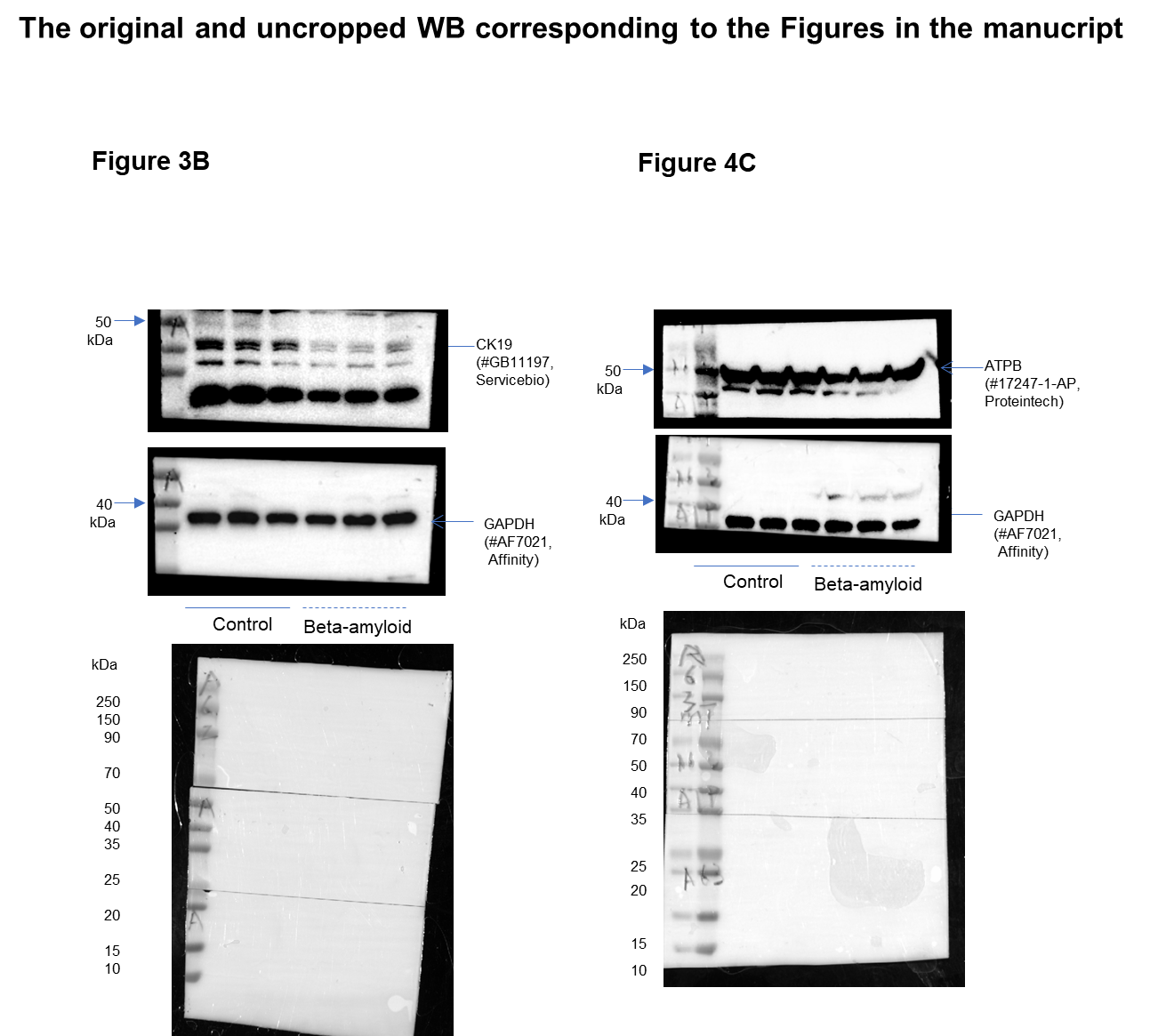


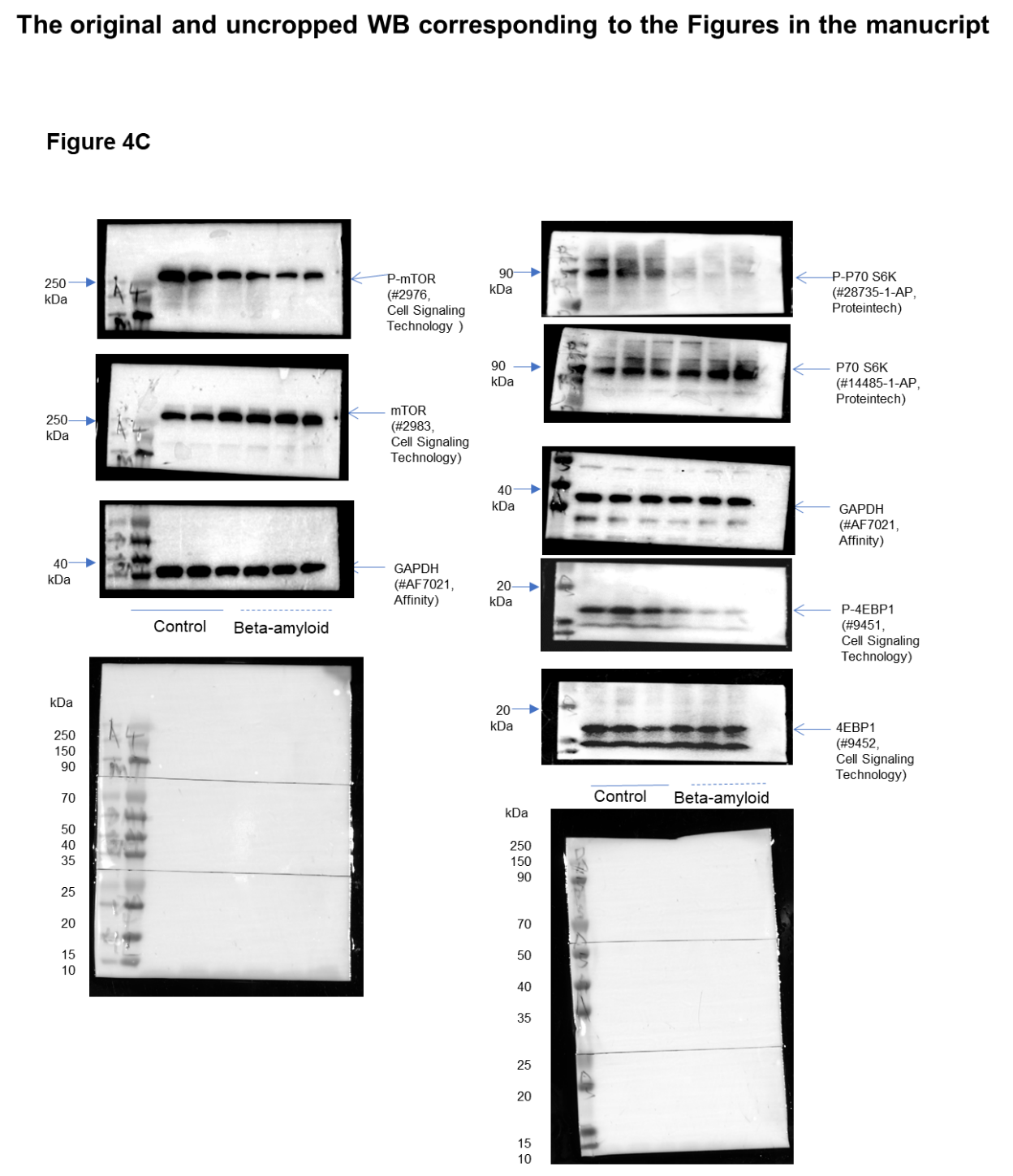


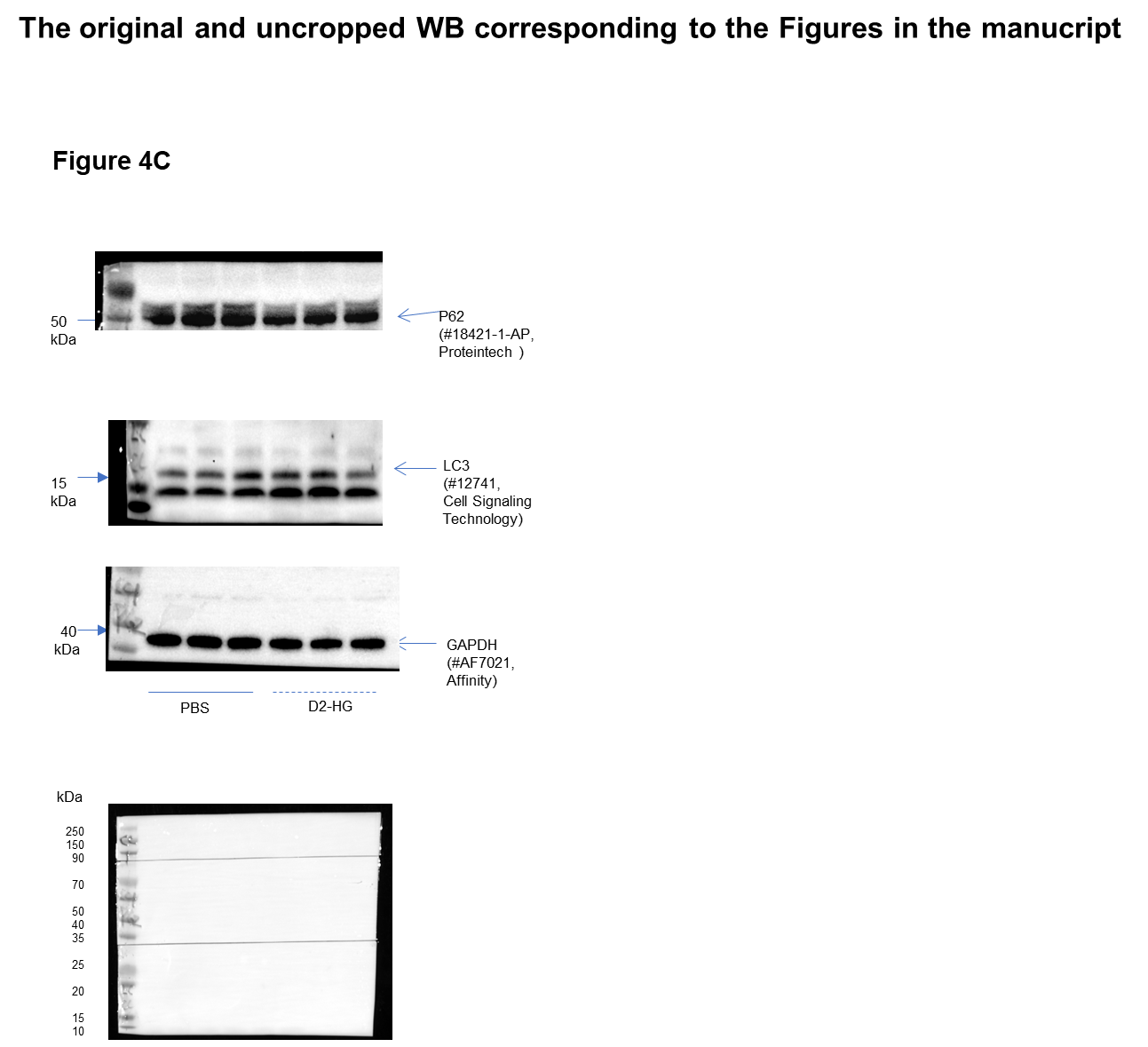

Supplement: Supplementary file 1 [file ct9-13-e00536-s001.docx]
